# Inappropriate interpretation of non‐pathogenic *HTRA1* variant as pathogenic

**DOI:** 10.1002/acn3.51817

**Published:** 2023-05-31

**Authors:** Masahiro Uemura, Sho Kitahara, Taisuke Kato, Hiroaki Nozaki, Shoichiro Ando, Tomohiko Ishihara, Osamu Onodera

**Affiliations:** ^1^ Department of Neurology Brain Research Institute, Niigata University Niigata Japan; ^2^ Department of Molecular Neuroscience Brain Research Institute, Niigata University Niigata Japan; ^3^ Department of Medical Technology, Graduate School of Health Sciences Niigata University Niigata Japan

Letter to the editor

Recently, novel *high‐temperature requirement A serine peptidase 1 (HTRA1)* mutations have been identified as a cause of cerebral small vessel disease (CSVD) in increasing numbers of patients. Zhang et al. reported eight *HTRA1* variants, including A20V (rs369149111), as causative mutations for CSVD in a sample of 181 Chinese patients.^1^ While they demonstrated a loss‐of‐function property for A20V using in vitro assay, we have several concerns about this study.

First, A20V is a common variant, particularly among East Asians, with an allele frequency of 0.1480 according to the gnomAD browser (https://gnomad.broadinstitute.org/) and 0.2112 among Japanese individuals according to dbSNP (https://www.ncbi.nlm.nih.gov/snp/). To determine the pathogenicity of such common variants, it is necessary to investigate whether the clinical features or frequency of A20V carriers differ significantly between CSVD patients and the general population. Second, previous studies have shown that the location of the variant can strongly influence the protease activity of HTRA1.^2^ Variants located outside the linker region or protease domain are unlikely to affect protease activity.

Therefore, we retrospectively investigated the frequency of A20V carriers in our database of CSVD patients. Clinical information and blood samples were collected from neurological centers in Japan. Patients with severe white matter hyperintensity corresponding to Fazekas grade 3/III and an age of onset of neurological symptoms/signs ≤70 years were included. After purifying genomic DNA, genetic testing for *NOTCH3* and *HTRA1* was performed, and whole exome sequencing was additionally performed if hereditary CSVD was strongly suspected. After excluding patients with causative mutations for CSVD, we identified 13 heterozygous A20V carriers among 62 CSVD patients (21.0%). No homozygous mutations were detected in this study.

Next, we investigated the protease activity of A20V. Expression plasmids for wild‐type (WT), A20V, and S328A complementary DNA were generated. Because A20V is located in the signal peptide,^3^ we also examined whether the A20V variant influenced the secretion of the HTRA1 protein. Culture supernatants containing overexpressed HTRA1 proteins after transfection into HEK293T cells were used to measure protease activity (Supplementary methods). The protease activity of A20V was not significantly different from that of WT HTRA1 (Fig. 1) and insufficient secretion of the A20V variant was not detected.

**Figure 1:**
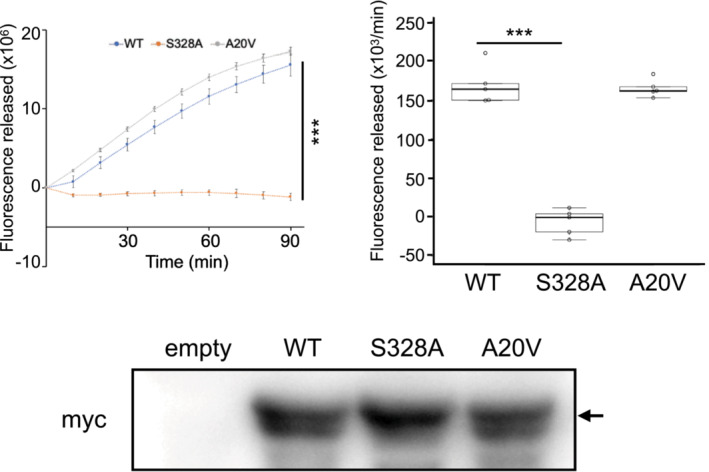
Protease activity of A20V variant HTRA1 protein. (Upper left panel) The fluorescent values were plotted against each time points. The graph shows the values from one independent experiments of five samples of each proteins are shown. WT and S328A indicates the positive and negative controls, respectively. I bars indicate standard errors. The fluorescent values at 90 min were statistically compared with one‐way analysis of variance followed by the Bonferroni correction. ***p‐value < 0.0001 for protease activities of each HTRA1 relative to WT. (Upper right panel) The boxplots and swarmplots show the protease reaction rate calculated from the slope of the linear portion of the fluorescence versus time plots. Protease reaction rate was statistically compared with one‐way analysis of variance followed by the Bonferroni correction. ***p‐value < 0.0001 for protease activities of each HTRA1 relative to WT. (Lower panel) SDS–PAGE of empty vector, WT, S328A, and A20V variant HTRA1 proteins used in the protease assay. Black arrows indicate the full‐length band of HTRA1 tagged with myc‐His6.

Collectively, these results suggested that the A20V variant is not pathogenic for CSVD. Inappropriate interpretation of *HTRA1* variants may unnecessarily cause anxiety in patients. A thorough assessment including epidemiological and biological factors is necessary for the identified *HTRA1* variants.

## Author Contributions

*Conceptualization; Data curation; Formal analysis; Investigation; Methodology; Writing – original draft; Writing – review & editing*: Masahiro Uemura. *Investigation*: Sho Kitahara. *Methodology; Investigation*: Taisuke Kato. *Genetic testing; Obtaining patient information; Writing – review & editing*: Hiroaki Nozaki. *Data curation; Writing – review & editing*: Shoichiro Ando. *Writing – review & editing*: Tomohiko Ishihara. *Conceptualization; Data curation; Formal analysis; Funding acquisition; Resources; Supervision; Writing – review & editing*: Osamu Onodera.

## Funding Information

The study was funded by a grant‐in‐aid for Scientific Research on Innovative Areas (Brain Protein Aging and Dementia Control; 26117006) from MEXT; a grant‐in‐aid for Practical Research Project for Rare/Intractable Diseases (19ek0109236h0003) from AMED; a grant‐in‐aid for Scientific Research (A) (19H01043); a grant‐in‐aid for Medical Research from the Takeda Science Foundation; and a grant‐in‐aid for Research on Intractable Disease (21FC0201) from the Japanese Ministry of Health, Labor and Welfare, Japan.

## Conflict of Interest Statement

Osamu Onodera is a speaker honorarium for Kyowa Hakko Kirin, Bristol‐Myers Squibb, Ono Pharmaceutical, Mitsubishi Tanabe Pharm, Takeda, Daiichi‐Sankyo, FUJIFILM, SANOFI, FP‐pharm. The other authors have no conflicts of interests to declare.

## Supporting information


**Data S1** Supporting Information.
