# Supplementary material for: Inappropriate interpretation of non‐pathogenic HTRA1 variant as pathogenic
Source: Ann Clin Transl Neurol. 2023 May 31;10(7):1261–2. doi: 10.1002/acn3.51817 (PMC10351663; doi:10.1002/acn3.51817)
Supplement: Supplementary file 1 — Data S1 Supporting Information. [file ACN3-10-1261-s001.docx]

**Title:**

**Inappropriate interpretation of non-pathogenic *HTRA1* variant as pathogenic**

Running title:

Reporting non-pathogenic HTRA1 variant as a cause

Authors:

Masahiro Uemura, MD, PhD^1)^, Sho Kitahara, MD^1)^, Taisuke Kato, MD, PhD^2)^, Hiroaki Nozaki, MD, PhD^3)^, Shoichiro Ando, MD, PhD^1)^, Tomohiko Ishihara, MD, PhD^1)^, and Osamu Onodera, MD, PhD^1)^.

Affiliations:

1. Department of Neurology, Brain Research Institute, Niigata University, Niigata, Japan
2. Department of Molecular Neuroscience, Brain Research Institute, Niigata University, Niigata, Japan
3. Department of Medical Technology, Graduate School of Health Sciences, Niigata University, Niigata, Japan

Address correspondence to: Osamu Onodera MD, PhD

Department of Neurology, Brain Research Institute, Niigata University, Niigata, Japan.

1-757, Asahimachi-dori, Chuoku, Niigat 951-8585, Japan

Tel: +81-25-227-0666; Fax: +81-25-223-6646

E-mail: onodera@bri.niigata-u.ac.jp

Submission type: Letter to the editors

Figures 1; Tables 0

Number of references: 3

Title: 66 ≤ 100 characters

Abstract: Not available

Main text: 393 (≤ 400 words)

**Supplementary methods**

**Vectors for human HTRA1 expression**

Expression vectors were designed to express wild-type and mutant HTRA1 as a fusion protein with a C-terminal myc/His6 tag under the control of the CMV promoter. The S328A, which is artificially and effectively inactivate enzyme activity, were utilized as mutant forms of HTRA1 in this study.

**Cell culture**

Human embryonic kidney (HEK)293T cells were cultivated in DMEM medium supplemented with 10% FBS. Equal amounts of HTRA1 expression vectors or empty vectors lacking *HTRA1* cDNA were transfected into HEK293T cells using lipofectamine 3000 (Thermo Fisher Scientific). Culture supernatants were collected for protease assay and immunoblotting 48 hours following transfection.

**Measurement of HTRA1 protease activity**

Equal volumes of culture supernatants containing overexpressed human HTRA1 proteins were incubated with fluorescein isothiocyanate (FITC)-labeled casein (Thermo Fisher Scientific) as a substrate at 37℃. Fluorescence was measured using a FilterMax F5 Multi-Mode Microplate Reader (Molecular Devices). Normalized released fluorescent values was calculated by subtracting the values measured in the blank empty vector sample from those in the sample containing overexpressed recombinant HTRA1 at each time point. Additionally, the reaction rate was determined from the slope of the plot of fluorescence values versus time at 30, 60 and 90 minutes, with the slope of the plot indicative of a linear relationship^1^.

**Immunoblotting**

Equal volumes of culture supernatants containing overexpressed human HTRA1 proteins were subjected to SDS-polyacrylamide gel electrophoresis and transferred to a PVDF membrane. The membranes were probed with antibodies directed against the myc tag and βactin. Immunoreactivity on the membranes was detected through the use of peroxidase-conjugated secondary antibodies followed by a chemiluminescence reaction.

**Statistics**

Mean values were utilized for statistical analysis. Data comparison was statistically analyzed using one-way analysis of variance (ANOVA), followed by Bonferroni's post hoc test. A p-value of < 0.05 was set as the threshold for statistical significance. Statistical analysis was conducted by using Matlab 2021a Update 5 (9.10.0.1739362).

**References**

1. Uemura M, Nozaki H, Koyama A, et al. *HTRA1* mutations identified in symptomatic carriers have the property of interfering the trimer-dependent activation cascade. Front Neurol. 2019;10:693.
